# Mind how you cross the gap! Outcomes for young people who failed to make the transition from child to adult services: the TRACK study

**DOI:** 10.1192/pb.bp.115.050690

**Published:** 2016-06

**Authors:** Zoebia Islam, Tamsin Ford, Tami Kramer, Moli Paul, Helen Parsons, Katherine Harley, Tim Weaver, Susan McLaren, Swaran P. Singh

**Affiliations:** 1 Leicestershire and Rutland Hospice, Leicester; 2 University of Exeter Medical School, Exeter; 3 Imperial College London; 4 University of Warwick, Coventry; 5 South London and Maudsley NHS Foundation Trust; 6 Middlesex University, London; 7 London South Bank University

## Abstract

**Aims and method**

The Transitions of Care from Child and Adolescent Mental Health Services to Adult Mental Health Services (TRACK) study was a multistage, multicentre study of adolescents' transitions between child and adult mental health services undertaken in England. We conducted a secondary analysis of the TRACK study data to investigate healthcare provision for young people (*n* = 64) with ongoing mental health needs, who were not transferred from child and adolescent mental health services (CAMHS) to adult mental health services mental health services (AMHS).

**Results**

The most common outcomes were discharge to a general practitioner (GP; *n* = 29) and ongoing care with CAMHS (*n* = 13), with little indication of use of third-sector organisations. Most of these young people had emotional/neurotic disorders (*n* = 31, 48.4%) and neurodevelopmental disorders (*n* = 15, 23.4%).

**Clinical implications**

GPs and CAMHS are left with the responsibility for the continuing care of young people for whom no adult mental health service could be identified. GPs may not be able to offer the skilled ongoing care that these young people need. Equally, the inability to move them decreases the capacity of CAMHS to respond to new referrals and may leave some young people with only minimal support.

Nationally^1,2^ and internationally^3,4^ policy makers and service providers have stressed that young people should have access to appropriate mental health services as they grow into adulthood. UK services for teenagers are split between child and adolescent mental health services (CAMHS) and adult mental health services (AMHS). The time point when a young person transfers to AMHS differs according to local service design (e.g. locality *v*. regional, generic *v*. diagnosis-specific services)^5,6^ and can lie between the ages of 16 and 18 years.^6^

As adult services focus on severe mental illness,^7^ young people with other ongoing mental health disorders, such as emotional, neurodevelopmental (e.g. autism spectrum disorder, attention-deficit hyperactivity disorder (ADHD)) or emerging personality disorders can fall through the CAMHS–AMHS gap.^8^ There is concern that these young people have poor outcomes (e.g. increased rates of attendance at accident and emergency departments, employment problems, contact with criminal justice and social care systems).^9,10^ They may present to adult services later, when in crisis or having developed severe and enduring mental health problems.^11–13^

The Transitions of Care from Child and Adolescent Mental Health Services to Adult Mental Health Services (TRACK) study was a multistage, multicentre study of adolescents' transitions between CAMHS and AMHS, undertaken in the English National Health Service (NHS). It included an audit of policies and procedures relating to transition,^6^ a case-note survey, an organisational analysis^13–16^ and a qualitative exploration of the views of patients, carers and mental health professionals on the process of transition.^17^ This paper provides further data and transition outcomes for young people with ongoing mental health needs who did not transfer to AMHS.

## Method

The full details of the TRACK methodology have been reported elsewhere.^13,18^ Briefly, from September 2003 in London and from January 2006 in the West Midlands, TRACK followed over 1 calendar year the journey of a prospectively identified cohort of young people who reached the service-specific transition boundary in six mental health trusts (providers of mental health services) within the NHS in England.^13,16^ These trusts covered a sociodemographically diverse population of 8.1 million in urban and rural areas and provided specialist (secondary care) mental health services, free at the point of delivery. Epidemiological studies suggest that the prevalence of impairing psychiatric disorder among young people at 8–18% of the school-age population with about half persisting into young adulthood,^19,20^ and that in Great Britain approximately 25% of school-age children with a disorder will access mental health services over the following 3 years.^21^ All specialist CAMHS teams that referred to local AMHS within these trusts were included. Highly specialist tertiary CAMHS (such as national centres) were excluded because of the atypical populations they served and the logistical problems created by their interfaces with AMHS from around the country, most beyond the participating trusts.

To identify CAMHS teams that met the inclusion criteria the local collaborators for each site were asked to identify services and set up face-to-face meetings with the lead clinician for each, who, in turn, were also asked to identify suitable teams. Within each included team, actual and potential referrals were identified from the preceding year using a two-stage process:

- phase 1 – central databases' searches
- phase 2 – asking individual clinicians within teams to identify actual and potential referrals in the preceding year.

The exact dates for the preceding year differed for each trust owing to data being collected at different time periods, but the data were collected from all sites for a 12-month period between 2005 and 2007.^13,18^

Following case identification, young people's journey from CAMHS, across the transition boundary and for up to 3 months following referral to AMHS was ‘tracked’ using a case-note survey. For this reason the term ‘cases’ rather than participants will be used throughout this paper. The data extraction tool used had been piloted for reliability, and included sociodemographic and clinical, transition pathway and transition outcome details.^18^

### Diagnoses

As the majority of CAMHS case records failed to record a diagnosis, presenting problems were identified and independently assigned by three CAMHS psychiatrists (M.P., T.F. and T.K.) to the following seven diagnostic groups:

1. serious and enduring mental disorders (including schizophrenia, psychotic disorders, bipolar affective disorder, depression with psychosis)
2. emotional/neurotic disorders (including anxiety, non-psychotic depression, post-traumatic stress disorder (PTSD), obsessive–compulsive disorder)
3. eating disorders (anorexia nervosa, bulimia nervosa, atypical eating disorder)
4. conduct disorders (including other behavioural disorders)
5. neurodevelopmental disorders (including autism spectrum disorder, ADHD, intellectual disability)
6. substance use disorders (alcohol and/or drug misuse)
7. emerging personality disorder.

Cases could be assigned to more than one category. Discrepancies in assignment to diagnostic categories were resolved through discussion.

### Cases tracked

As previously reported,^13,16^ CAMHS cases were tracked. One case was excluded from subsequent analysis as transition was to an adult neurology and not a mental health service. The sample consisted of 78 (51%) males and 76 females, with a mean age of 18.12 years (s.d. = 0.824) at the time of data collection. The majority ethnic groups were White (*n* = 47, 31%) and Black (*n* = 36, 23%), but no ethnicity was recorded for a large portion of the sample (*n* = 41, 27%). Other sociodemographic details of the cohort have previously been reported in Singh *et al*.^13^

Over four-fifths of the TRACK cohort cases were considered suitable for transfer by CAMHS (*n* = 131, 85.1%), but over a third of these (*n* = 52, 40.0%) were not referred by CAMHS to any AMHS. Of those who were referred, 3 cases were pending a decision from AMHS and 20 cases (13.0%) had at least one AMHS referral rejected at the cessation of data collection. Of those initial 20, 11 (7.1%) were successfully accepted by another AMHS and 2 had decisions pending at the point of data collection, leaving 7 (11%) who were unsuccessfully referred to AMHS.

Overall, there were 64 young people with ongoing mental health needs who were not transferred to AMHS and who form the basis of the current analysis: 5 cases (8%) were pending a decision from AMHS at the point of data collection, 7 (11%) were unsuccessfully referred and the remaining 52 (81%) were not referred to AMHS by CAMHS.

### Analysis

This secondary analysis focuses on the ‘potential cases’: those young people who were not accepted by AMHS, regardless of the reason of the failed transition. Young people who are not potential cases (‘actual cases’) are those in the TRACK cohort who successfully crossed the transition boundary from AMHS and were accepted by an AMHS.

After the potential cases were identified from the TRACK database, variables of interest were re-coded to allow for comparison between the three different types of potential cases. Where multiple options were recorded categories were combined (e.g. carer or young person refused AMHS referral) or a new category created (e.g. comorbid diagnosis; multiple reasons for no referral to AMHS), depending on frequency of category use.

## Results

### Diagnostic categories

The majority of the 64 cases with ongoing mental health needs not transferred to AMHS were young people with emotional/neurotic disorders (*n* = 31, 48.4%). It is alarming that two young people recorded as having serious and enduring mental illness were among this group. Others had neurodevelopmental disorders (*n* = 15, 23.4%); comorbidity (*n* = 6, 9.4%); an eating disorder or no recorded diagnosis (*n* = 4, 6.3% each); or conduct disorder and substance misuse (*n* = 1, 1.6% each).

### Unsuccessful referrals to AMHS

**Fig. 1:**
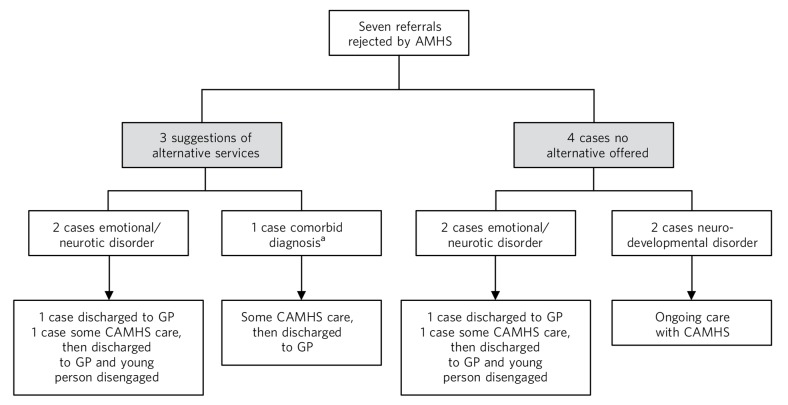
The outcomes of unsuccessful transitions. AMHS, adult mental health services; CAMHS, child and adolescent mental health service; GP, general practitioner. a. Emotional/neurotic and neurodevelopmental disorders.

As Fig. 1 illustrates, three out of the seven unsuccessful referrals were offered alternative services by the rejecting AMHS; all had a diagnosis of emotional/neurotic disorder but one also had a comorbid neurodevelopmental disorder. One case did not list the alternative service, one case suggested crisis support and one case was suggested referral to community mental health services. All three cases were subsequently closed by CAMHS and referred back to primary healthcare, i.e. their general practitioner (GP). Of the remaining four young people who were not signposted to other services, two had a diagnosis of emotional and neurotic disorder; their cases were closed by CAMHS and they were discharged to their GP. The other two young people had diagnoses of neurodevelopment disorder and continued to receive care from CAMHS beyond the transition boundary, as an alternative service could not be found.

### Cases referred to AMHS and pending AMHS decision

**Fig. 2:**
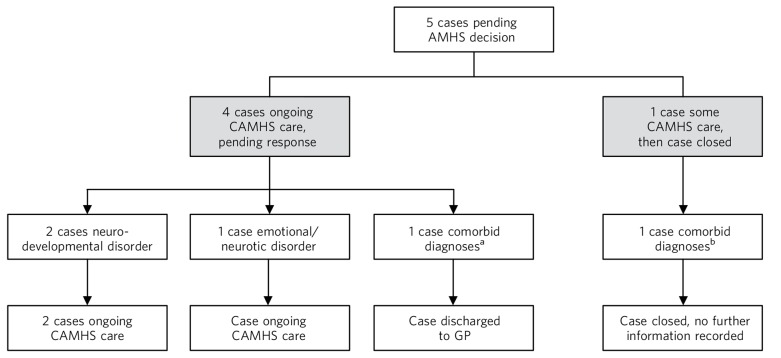
Outcomes of pending cases. AMHS, adult mental health services; CAMHS, child and adolescent mental health service; GP, general practitioner. a. Dual diagnosis of conduct and neurodevelopmental disorders. b. Dual diagnosis of emotional/neurotic and neurodevelopmental disorders.

Figure 2 shows that of the five pending cases, one was subsequently closed by CAMHS without any subsequent care. This young person had a dual diagnosis of conduct and neurodevelopmental disorders, but no information about further care was recorded. Three cases were still open to CAMHS at the time data collection was completed. One case, with an emotional/neurotic disorder, had received ongoing care for 46 weeks beyond the transition cut-off age; one case with a neurodevelopmental disorder had received ongoing care for 7.6 weeks at the time data collection was completed. The third open case also had a diagnosis of neurodevelopmental disorder, and although it was marked as not closed at the time of completion of data collection, there was no record of how long ongoing care had been received at CAMHS after the referral was made. The final pending case was of a person with dual diagnosis of emotional/neurotic and neurodevelopmental disorders. Although this case had not been formally closed by CAMHS, care was not continued and the young person was effectively discharged to their GP.

### Cases that were not referred

**Fig. 3:**
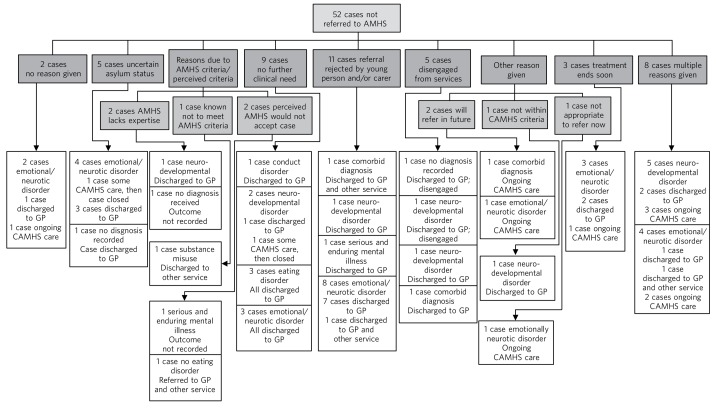
Outcomes of potential referrals. AMHS, adult mental health services; CAMHS, child and adolescent mental health service; GP, general practitioner.

The outcomes of the remaining 52 potential cases are illustrated in Fig. 3. The most commonly cited reason for CAMHS not making a referral to an AMHS was that the referral was refused by the young person, their carer or both parties (*n* = 11 cases, 21% of not referred cases). The next most commonly cited reasons were that, by then, there was no further clinical need for treatment (*n* = 9, 17%) or that the young person had disengaged from services (*n* = 5, 10%). The remaining cases (*n* = 8, 15%) were not referred either because:

- cases were assumed not to meet AMHS referral criteria and AMHS were perceived by CAMHS not to have the required expertise (in one of these three cases the young person was pregnant) or because the immigration status of the young person was uncertain (one case); the reason was not recorded in one case
- the plan was to refer later as: the immigration status of the young person was uncertain at the time (one case), the young person was in prison (one case), referral was being refused by the young person and parent/carer at the time (one case) or no reason recorded (one case).

The most common healthcare outcomes were discharge to GP (*n* = 29, 56%) and ongoing CAMHS care (*n* = 13, 25%). Four cases (8%) had multiple outcomes recorded:

- one was reported to have some ongoing CAMHS care, discharged to GP, disengaged, which implies that adults were worried about the young person and trying to re-engage them
- some ongoing CAMHS care then closed by CAMHS, discharged to GP and other service (asylum services), lost to follow-up
- discharged to GP and alcohol recovery service
- discharged to GP and other service (not stated).

Emotional/neurotic disorder was the most common recorded diagnosis among both those who were discharged to their GP (*n* = 14, 54% of this diagnostic group) and young people who continued to have support from CAMHS (*n* = 9, 35% of diagnostic group). Neurodevelopmental disorders were the next most common diagnoses among young people with both outcomes (*n* = 7, 64% of those referred to their GP; *n* = 3, 27% of diagnostic group with continuing care from CAMHS).

## Discussion

Previous papers reporting TRACK findings have highlighted that AMHS accepted 93% of referrals they received^18^ and that the main reasons for non-referral to AMHS were refusal by adolescents or parents/carers, CAMHS clinicians thinking AMHS would not accept the referral/that AMHS had no appropriate service or that CAMHS were still planning to refer to AMHS.^16^ This paper focuses on transition outcomes for the significant proportion of cases (34% of cohort) who had not been referred to AMHS on reaching the transition boundary, despite having ongoing mental health needs. These cases typically had neurodevelopmental or emotional/neurotic disorders, highlighting that young people with these disorders are those most likely to fail to access secondary healthcare. Failure to transfer to AMHS resulted most commonly in discharge to a GP, raising questions about the extent to which their ongoing needs would be met by primary care. It is unclear whether GPs have sufficient training or expertise to manage or deliver mental healthcare to young adults presenting in this way.^22^

Over half of those who did not transfer to AMHS, however, continued to receive some CAMHS care after crossing the transition boundary, with a large number of these cases yet to be closed by CAMHS at the time of completing data collection, i.e. at least 3 months following crossing the transition boundary. This finding has implications for the capacity of CAMHS, which often struggle to meet the demand for new referrals, resulting in long waiting lists for assessment or treatment.^23^

Although a small proportion of cases were referred to another service, this was often in tandem with referral to primary care. Additional services, such as asylum services, were not always focused on the mental health needs of the young person. The most common reason for CAMHS not making a referral to AMHS was rejection of the referral by the young person and/or their carer, followed by the resolution of clinical need. The latter might reflect a plan to complete an episode of care, for instance finishing a course of cognitive–behavioural therapy, and seems an appropriate outcome. The next most common reason was CAMHS failing to refer to AMHS. CAMHS practitioners can come from a multitude of disciplines, each with different training and perspectives, which may lead to different conceptualisations of the same child's difficulty and different levels of competence in assessment and formulation or diagnosis.^24^ In some quarters, there is particular antipathy towards ‘the medical model’; psychiatrists in particular face criticism for the perception that they apply narrowly focused ‘disease’ concepts to psychopathology.^25,26^ It may be that cultural differences between the conceptualisation of difficulties between practitioners working in CAMHS and AMHS, particularly those from a non-medical background, may lead to a failure in communication that undermine efforts to transfer the care of young people as they become adults. Although data were not collected on this aspect, it is possible that CAMHS clinicians may quite appropriately not refer to AMHS because of knowledge and prior experience of local AMHS referral criteria and patterns of refusal of referrals. This remains a hypothesis to be tested by research, however, TRACK organisational findings suggest that working cultures, lack of clarity on service availability and eligibility issues can also influence transition.^14,15^ Ideally, CAMHS clinicians should refer to AMHS in order to document the need for provision and better commissioning for these young adults. Retaining cases in CAMHS also risks young adults disengaging from services that are not age appropriate as well as reducing the capacity of CAMHS to respond to new referrals. It suggests gaps in service provision discussed below, particularly for young people with emotional or neurodevelopmental disorders.

Although there has been increasing acknowledgement by many professionals that ADHD persists into adulthood, many adult mental health professionals remain sceptical about the validity of ADHD as a true disorder and in particular as a disorder that affects adults.^27–30^ The UK National Institute for Health and Care Excellence (NICE) ADHD guideline group concluded that ADHD is a valid disorder that continues into adulthood and that adults with ADHD should be identified and managed within the NHS.^31^ At the moment, however, health services research would suggest that many adults with ADHD are refused services by adult mental health teams as ADHD is perceived as falling outside their remit or expertise. Where services do not exist, notably those for young people with neurodevelopmental disorders, unmet needs should be systematically documented and made clear to AMHS providers and commissioners.

Since our study was conducted, there has been expansion of relevant alternative provisions for emotional disorders such as Increasing Access to Psychological Therapies (IAPT) services or integrated disability services, accessed through social care. For adults, IAPT provides additional capacity to deliver psychological therapy for affective disorders. IAPT post-dates our data and so if we re-ran our study now, young adults with emotional and neurotic disorders might have more options once they turn 18. Equally, it should be noted that the time taken for referrals entering into such services can be lengthy and vary by region.^32^

Additionally, although third-sector (voluntary and independent) organisations might be commissioned or available to young adults with mild to moderate mental health problems or disorders, we found little third-sector service use in this case study. Future research should clarify to what extent these newer services have taken up cases that are graduating from CAMHS. It is clear, however, that intervention for some conditions, such as ADHD requiring medication,^33^ would not be provided by these services.

The TRACK study in the UK was the first systematic attempt to understand the policy, process, outcome and experience of transition from CAMHS to AMHS. The population studied was large and diverse, making findings generalisable to other services in the UK. The finding of this paper – that GPs and CAMHS are being given responsibility for the continuing care of young people for whom no AMHS can be identified – should be of interest to those working in different service structures nationally and internationally.

### Limitations

Limitations of the study include the small identified cohort, difficulties identifying cases and problems with data collection largely owing to inadequate CAMHS databases at the time of the study, as reported previously.^13,16^ Case notes may not have accurately reflected the quality and content of service provision or decision-making. Despite these issues, even if every case missing from this cohort had an ideal outcome, we have documented a large quantity of cases with likely unmet mental health needs after leaving CAMHS. The data-set is also a few years old and, since implementation of the Health and Social Care Act 2012 in England, ‘any qualified provider’ can now be commissioned to provide services. There is also a move towards provision of youth services^4^ and ‘0–25’ mental health services^34^ in some areas, but these are not yet widespread.The cases we see in CAMHS may be the cases seen in future years in adult services and new computerised notes (e.g. RiO electronic patient records) may demonstrate this. A future study exploring AMHS data could potentially detect how many cases have been known to CAMHS.

In the next 10 years, mental health problems are expected to increase among children and young people, with current predictions estimating at least a 50% increase in incidence rates.^35^ According to the US Department of Health and Human Services, by 2020 mental illness will be one of the five most common causes of morbidity, mortality and disability among children and young people.^36^ Loss of cases at the transition boundary constitutes a risk of ongoing and deteriorating mental health issues. The break in service between CAMHS and AMHS occurs at a key life stage; choices about education, occupation and childbearing during the teenage years can have a profound impact on subsequent life chances, whereas behaviours that predicate future health, such as diet, exercise, sexual activity and psychoactive substance use, develop during adolescence.^37^ Successful transition is important to facilitate recovery alongside mental health promotion and mental illness prevention in those vulnerable to ongoing mental health need. It also has the potential for future cost savings: the presence of mental illness during childhood and adolescence leads to ten times higher costs during adulthood.^38^

Overall, this study demonstrated that GPs and CAMHS have been left with the responsibility for the continuing care of young people for whom no AMHS could be identified. This decreases the capacity of CAMHS to respond to new referrals and may leave some young people with only minimal support on leaving CAMHS. Further research is required to show whether recent changes in commissioning, post-implementation of the Health and Social Care Act 2012, have plugged the gap for young people who do not meet the criteria for AMHS or who refuse referral but leave CAMHS with ongoing mental health needs.
